# Effects of the tailored activity program (TAP) on dementia-related symptoms, health events and caregiver wellbeing: a randomized controlled trial

**DOI:** 10.1186/s12877-021-02511-4

**Published:** 2021-10-20

**Authors:** Laura N. Gitlin, Katherine Marx, Catherine Verrier Piersol, Nancy A. Hodgson, Jin Huang, David L. Roth, Constantine Lyketsos

**Affiliations:** 1 Drexel University, Philadelphia, USA; 2 Johns Hopkins University, Baltimore, USA; 3 1601 Cherry Street, Suite 1092, PA 19102 Philadelphia, USA; 4 Thomas Jefferson University, Philadelphia, USA; 5 University of Pennsylvania, PA Philadelphia, USA

**Keywords:** Behavioral symptoms, Family caregiving, Tailored activities, Quality of life, Nonpharmacological strategies

## Abstract

**Background:**

People living with dementia (PLWD) and caregivers are adversely impacted by lack of meaningful activity leading to worse symptoms and impaired quality-of-life. There is a critical need to develop effective and well-tolerated treatments that mitigate clinical symptoms, engage PLWD and support caregiver wellbeing. We tested whether, compared to attention control, the Tailored Activity Program (TAP) reduced clinical symptoms and health-related events, and improved caregiver wellbeing, and if TAP activities were well-tolerated.

**Methods:**

We conducted a single-blind randomized controlled trial among 250 dyads recruited from Baltimore-Washington DC (2012–2016) with a dementia diagnosis and clinically significant agitation/aggression. Dyads were randomized to TAP (*n* = 124) or attention control (*n* = 126), and interviewed at baseline, 3 (endpoint) and 6-months (follow-up) by interviewers masked to group allocation. TAP assessed PLWD abilities/interests, instructed caregivers in using prescribed activities, and provided dementia education and stress reduction techniques. Attention controls received disease education and home safety tips. Both groups had up to 8 home visits over 3-months. The primary outcome was frequency by severity scores for agitation/aggression subscales of Neuropsychiatric Inventory-Clinician using caregiver ratings. Secondary outcomes included number of instrumental (IADL) and activities of daily living (ADL) needing assistance, caregiver wellbeing, and confidence using activities. Health-related events (PLWD death, hospitalizations, caregiver hospitalization, depression) and perceived study benefits were captured over 6 months. PLWD tolerability of prescribed activities was examined.

**Results:**

Of 250 dyads, most caregivers were female (81.2 %, *n* = 203), non-spouses (54.4 %, *n* = 136), white (59.2 %, *n* = 145) or African American (36.7 %, *n* = 90) with mean age = 65.4 (SD = 12.6). PLWD were mostly female (63.2 %, *n* = 158) with mean age = 81.4 (SD = 7.9), and mean MMSE = 14.3 (SD = 7.8). At 3-months, compared to controls, TAP conferred no benefit to agitation/aggression (*p* = 0.43, *d* = 0.11), but resulted in less IADL (*p* = 0.02, *d*=-0.33), and ADL (*p* = 0.04, *d*=-0.30) assistance, improved caregiver wellbeing (*p* = 0.01, *d* = 0.39), and confidence using activities (*p* = 0.02, *d* = 0.32). By 6-months, 15 PLWD in TAP had ≥ 1 health-related event versus 28 PLWD in control, demonstrating 48.8 % improvement in TAP (*p* = 0.03). TAP caregivers were more likely to perceive study benefits. Prescribed activities were well-tolerated.

**Conclusions:**

Although TAP did not benefit agitation/aggression, it impacted important outcomes that matter to families warranting its use in dementia care.

**Clinical trial registration:**

Clinicaltrials.gov # NCT01892579 at https://clinicaltrials.gov/; Date of clinical trial registration: 04/07/2013; Date first dyad enrolled: 15/11/2013.

## Introduction

Dementia is a worldwide public health challenge affecting over 50 million individuals and their families with this number expected to triple by 2050 [1]. The effect of dementia is profound impacting behavioral patterns, daily functioning, quality of life, activity engagement and health care utilization [2, 3]. Caregivers experience daily care challenges resulting in increased risk for depression, and poor quality of life [4]. A major cause of adverse outcomes for people living with dementia (PLWD) and caregivers are neuropsychiatric symptoms (NPS) which are almost universal and occur across all disease stages and etiologies [5]. Pharmacological options for NPS have modest benefits, while some are associated with significant risks including mortality, and do not typically address the many care challenges families identify as most problematic to them [6, 7]. Moreover, in the absence of effective pharmacotherapies that slow disease progression, there is an urgent need to develop and test the effectiveness of well-tolerated treatments that improve NPS and thereby enhance quality of life of PLWD and their caregivers [4].

Nonpharmacological approaches have been identified as first line treatments and have potential to address NPS as well as associated functional dependence, and caregiver distress [8, 9]. Although various nonpharmacological strategies have been tested, efficacy studies vary in quality, and results are inconsistent [10–12]. Moreover, most nonpharmacological approaches involve rapport building and individualized attention, yet few studies control for potential effects of attention on outcomes [13] .

An emerging evidence base suggests that activity engagement can help PLWD by reducing one of the most serious forms of NPS, agitation/aggression [11, 12, 14, 15]. Agitation/aggression-type behaviors can occur at any stage of the disease or with any etiology, but are particularly acute at the moderate stage when more hands-on care by family members are typically required. Lack of meaningful activity is also an expressed unmet need among PLWD and caregivers [16–18]. In previous trials, we showed that the Tailored Activity Program (TAP), which tailors activities to abilities and interests and instructs caregivers in their use in addition to providing disease education and stress reduction techniques, resulted in fewer NPS, reduced functional dependence, and enhanced caregiver wellbeing [15, 19–22]. However, it is unclear from these previous trials whether personalized attention contributes to or accounts for positive NPS and other favorable outcomes [14, 15]. Also unclear is whether disease education and support alone would reduce agitation/aggression.

The objective of this phase III randomized clinical trial was to test the effectiveness of TAP to reduce agitated and aggressive behaviors, two of the most prevalent and upsetting behavioral symptoms of dementia [23] for caregivers and PLWD. We hypothesized that participation in TAP compared to an attention control would result in a reduction in agitated/aggressive behaviors at three months. We also sought to determine if TAP impacted other outcomes that matter to PLWD and caregivers including functional dependence, health-related events, and caregiver wellbeing and confidence using activity as a care strategy. We hypothesized that PLWD in comparison to those in attention control would experience less functional dependence at three months, and fewer health-related events (death, hospitalizations, emergency room visits, and depression and/or suicidal ideation as reported by their caregivers) by six months. Similarly, we hypothesized that caregivers in TAP compared to attention control would report better overall well-being and confidence using activities as part of daily care at three months, and fewer health-related events (hospitalizations, elevated depression scores) by six months. We also compared caregiver perceived study benefits at six months with the expectation that TAP caregivers would more likely report benefits than attention control caregivers. Finally, we evaluated whether activities prescribed in TAP were tolerated by PLWD when used by caregivers during the three-month intervention period.

## Methods

We report on a single blind two-armed randomized trial conducted between 2012 and 2016 in the Baltimore and Washington, DC regions. The trial was monitored by a data and safety monitoring board and overseen by a Johns Hopkins University Institutional Review Board. Written informed consent was obtained from caregivers and PLWD, or proxy if the PLWD was unable to consent [24]. Details of the study protocol have been described elsewhere [25]. All participants were assessed in their homes at baseline, 3 months (main study endpoint), and 6-months by trained research assistants masked to group allocation. All intervention sessions also occurred in homes. The trial adhered to CONSORT guidelines.

### Study population

Participants were recruited from the Baltimore region using various strategies including mailings to families by service providers, media announcements, talks at local community health seminars and events, and online trial searches (e.g., Alzheimer’s Association TrialMatch® and www.clinicaltrials.gov).

Eligibility criteria extended to both caregivers and PLWD. Dyads were eligible if the PLWD was English-speaking, had a physician’s diagnosis of dementia (mild, moderate, severe); was able to participate in at least two activities of daily living; and had agitated/aggressive-type behaviors. For the latter, caregivers had to endorse at least one behavior listed on the agitation and/or aggression domain of the Neuropsychiatric Inventory (NPI-C) with a frequency or severity score of ≥ 2 (moderate) [26]. If only one item on the agitation/aggression subscales was endorsed with a frequency < 2, then at least two other behaviors on the NPI-C had to be endorsed with a frequency or severity score ≥ 2. Eligibility was confirmed after review by a medical team (two Gero-psychiatrists and a nurse PhD gerontologist) to assure a sample with clinically meaningful neuropsychiatric symptoms. Additionally, as per best practice in clinical trials, if PLWD were on any of four classes of psychotropic medications (antidepressants, benzodiazepines, antipsychotics, or anticonvulsants) or an anti-dementia medication (memantine or cholinesterase inhibitor), a stable dose for 60 days was required prior to enrollment to minimize confounding effects of medications on outcomes.

Dyads were eligible if the caregiver was English-speaking; a family member (relative, neighbors, fictive kin), ≥ 21 years of age; lived with PLWD, or within 5 miles or 15 min driving time; accessible by telephone to schedule interview and intervention sessions; and planned to live in area for at least 6 months. As per best practice in clinical trials, caregivers taking a psychotropic medication (antidepressants, benzodiazepines, antipsychotics, or anticonvulsants), had to have been on a stable dose for 60 days prior to enrollment.

Dyads were excluded if PLWD had a previous psychiatric history (schizophrenia, bipolar disorder), dementia secondary to head trauma if unresponsive to their environment (e.g., unable to understand short commands or recognize a person coming in or out of the room) or if the caregiver was concurrently enrolled in another clinical trial or planned to place PLWD in a residential facility within 6 months. Finally, dyads were excluded if either had a terminal illness with life expectancy < 6 months, were in active cancer treatment, or had > 3 acute medical hospitalizations in the past year. Criteria were designed to minimize attrition due to poor health or exclude dyads who would not potentially benefit from study participation.

**Fig. 1:**
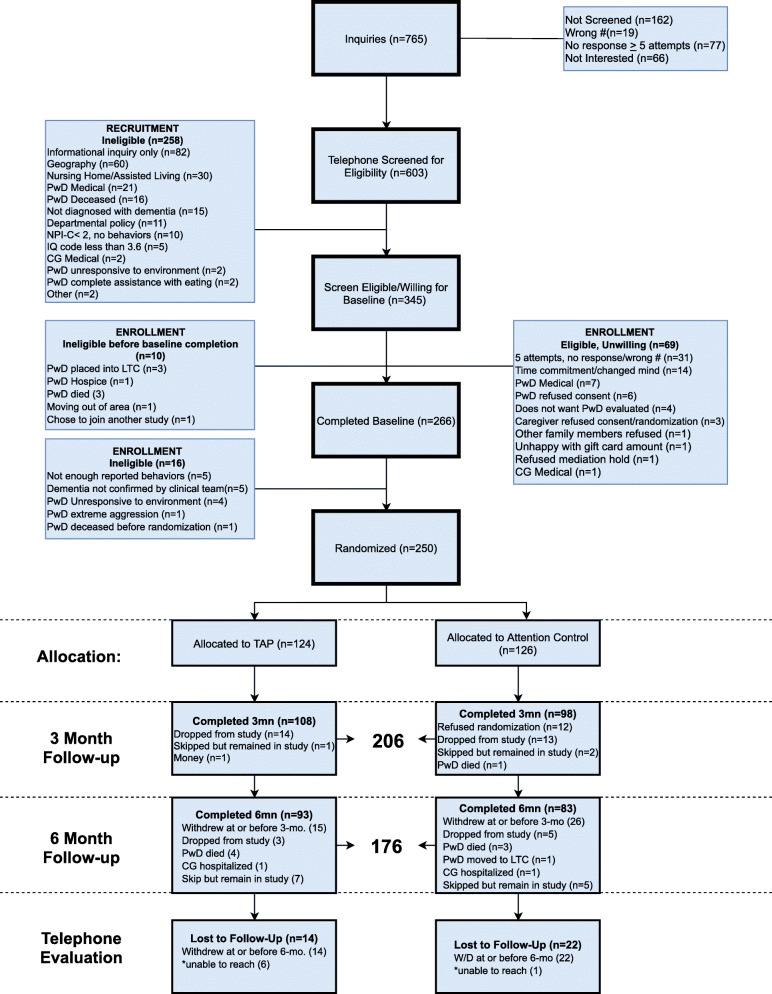
Consort Chart

Screening processes have been reported in detail elsewhere [25] and enrollment and retention results are presented in Fig. 1. Interested caregivers were initially screened by telephone with those preliminarily eligible having an in-home visit to obtain informed consent and conduct the baseline interview. (Figure 1) Following baseline interview, its review by the medical team and their final determination of eligibility, randomization occurred.

### Randomization

Participants were randomized 1:1 to TAP or an attention control group using a computer-based assignment scheme stratified by cognitive status using the Mini-Mental Status Examination (MMSE > 10 vs. MMSE ≤ 10) in randomized block sizes. Allocation concealment was protected by altering block sizes for stratification and using double opaque envelopes prepared by the statistician which were opened only by the project manager following completion of a baseline interview.

### Interventions

The Tailored Activity Program (TAP) has been described in detail elsewhere [25, 27, 28] Briefly, the program is delivered by occupational therapists (OT) and unfolds in three phases involving up to 8, 1 to 1½ hour sessions over three months. The first phase (up to two sessions), involves assessment of PLWD preserved abilities, functional challenges (fall risk, executive function, sensory impairments) and interests (roles, occupations, routines, activities), caregiver communication style, availability and readiness to use activities, and the physical environment (lighting, seating, noise, clutter). The assessment phase includes participation of caregivers and PLWD and yields an understanding of preserved abilities including areas of executive dysfunction from which strategies for engaging the PLWD in activities of intrinsic interest are formulated.

Based on assessments, the second phase, implementation, involves up to four sessions in which the OT identifies three specific activities of interest with each then tailored to PLWD functional, caregiver and environmental profiles. Caregivers are then instructed in their use including how to set-up an activity, communicate effectively, provide verbal or physical cues and/or supervise. Additionally, environmental adjustments (decluttering, seating, lighting) are implemented to support engagement in prescribed activities. To learn activity use, caregivers initially observe OTs providing each activity to PLWD while explaining the process and eliciting PLWD input. Then, caregivers try engaging PLWD in that activity as OTs observe, prompt and support the dyad. Caregivers are then asked to try the activity before the following session. Caregivers learn through observation and then through doing, and strategies are also provided in the form of written instructions, referred to as the TAP Activity Prescription for each prescribed activity. In each subsequent session, the activity previously introduced is reviewed and then a new activity prescription offered and practiced.

The third phase, generalization, involves up to two sessions with caregivers alone. In these sessions, OTs help caregivers generalize strategies from those learned for using activities to care challenges such as helping with bathing or dressing. For example, cueing techniques for supporting activity engagement may be able to assist PLWD in self-care. Simplifying the physical environment to support activity engagement can also be applied to everyday tasks such as bathing or grooming. Throughout TAP sessions, caregivers receive dementia education (e.g., behavioral symptoms are not intentional), and practice strategies and stress reduction techniques.

TAP is person-centered such that activities are designed based on interests and abilities of PLWD. As activities match interests, use familiar objects and are graded to functional profiles, activities provided differ vastly. Any one activity can be modified to fit the person’s cognitive functional abilities. For example, a PLWD with high cognitive status who enjoys gardening, may be able to engage in multiple steps such as digging a hole and planting flowers in their back yard (if not a fall risk). A PLWD with low cognitive status who previously enjoyed gardening, may be able to engage in a single step such as placing plastic flowers in a vase that is placed in their visual field.

In TAP, an activity can be designed for PLWD with MMSE scores ranging from 0 on up.

Examples of activities that were provided in this trial for those with low cognitive scores on the MMSE, sensory-type activities (music, balloon toss, watching nature/animal videos, using an activity board with objects of intrinsic interest, chair exercises); for those with moderate range scores, repetitive type activities (vacuuming, sorting coins or other objects, washing dishes, folding laundry, mixing salad ingredients); and for those with high cognitive status, multi-step activities (preparing a sandwich or salad; multi piece jigsaw puzzles, setting the table, dancing, walking, gardening).

OTs were trained through readings, a treatment manual, didactic and interactive teaching modalities, and bi-monthly supervision meetings. To monitor fidelity, all TAP visits were audiotaped of which 10 % were randomly selected for formal evaluation by staff who rated tapes using a score sheet with a priori criteria followed by feedback to the OT. At the conclusion of each session, OTs recorded the date and start and stop time for each session, if caregivers used activities between sessions and if so frequency of their use, and caregivers perceptions as to the level of PLWD engagement during the prescribed activity.

The attention control group, described in detail elsewhere [25], was designed to match TAP’s therapeutic engagement with caregivers. Delivered by trained research staff over 8 home sessions of up to 1 to 1½ hours each, caregivers received disease education, and home safety assessment and tips. However, sessions did not involve a systematic assessment of PLWD nor did caregivers receive education about NPS, functional decline, or an understanding of the abilities of PLWD or tailored activities, and stress reduction techniques, the active ingredients of TAP.

### Measures

Demographic characteristics included age in years, sex (male, female), race (white, African American, Latino, Asian, Native American, other), education (≥ high school, some college/associates, college degree/post-graduate degree), marital status (married/living as married, not married) and relationship to PLWD (spouse, non-spouse).

The primary trial outcome was frequency by severity scores of agitation/aggression behaviors at 3 months as measured by two domains of the Neuropsychiatric Inventory-Clinician version (NPI-C) [26]. For each of the 13 agitation subscale items, caregivers rated frequency (0 = never to 4 = very frequently). If frequency was greater than 0, then caregivers rated the severity (1 = mild to 4 = major source of distress). When frequency was 0, severity was set to 0. The 13-item agitation subscale had frequency and severity scores ranging from 0 to 52. For each of the 8-item aggression sub-scale items, caregivers similarly rated frequency and severity (range from 0 to 32). The primary outcome measure was obtained by multiplying the frequency rating times the severity rating for each item and adding these products across all 21 items on the two subscales with total scores potentially ranging from 0 to 336 (highest observed maximum score for this sample was 224), and higher scores reflecting greater frequency by severity.

Secondary outcomes included functional dependence, caregiver wellbeing and confidence using activities, and health-related events for PLWD and caregivers. The PLWD functional dependence in instrumental (IADL) and activities of daily living (ADL) was measured using the Caregiver Assessment of Function and Upset (CAFU) [29]. Caregivers rated eight IADLs (telephone, shopping, meal preparation, housework, laundry, travel, medicine, and finances), and seven ADLs (bathing, dressing upper body, dressing lower body, toileting, grooming, eating, getting in/out of bed) based on yes (1) PLWD needs assistance (supervision or hands-on) or no (0), PLWD did not need assistance. Totals for each area (ADL & IADL) were calculated with higher scores indicating more activities for which assistance was needed.

Caregiver well-being was measured with the 13-item Perceived Change for Better Index (α = 0.86), which assesses affective wellbeing, somatic, and ability to manage day-to-day care) along a five-point scale (1 = gotten a lot worse to 5 = improved). A total score was calculated by summing all 13 items, with higher scores indicating positive change [30].

Caregiver confidence using activities was measured with five items [14], from not at all confident (0) to very confident (10). Items included level of confidence: identifying daily or recreational activities PLWD is capable of doing; involving PLWD in daily and/or recreational activities; using activities to distract PLWD; using meaningful or pleasant activities to manage boredom, upset or agitation in PLWD; and setting up an activity (e.g. dressing, bathing, recreational activity) for PLWD. Items were summed (range 0–50) and an average score over the five items derived. Higher scores indicate greater caregiver confidence using activities with PLWD.

Number and type of health-related events were captured over 6-months by interviewers and interventionists. For PLWD, four events were followed: death, hospitalization, emergency room visits, and depression and/or suicidal ideation as reported by their caregivers. For caregivers, two events were followed: hospitalization and elevated depression scores (PHQ-9 ≥ 15) and/or suicidal ideation [31]. Depression was captured using the PHQ-9, a brief, psychometrically sound 9-item self-report measure which aligns with DSM diagnostic categories. To examine group differences at baseline, a mean total severity score was calculated by summing responses across the nine items which were rated as occurring not at all (0), several days (1), more than half the days (2), nearly every day (3). For this study, we were interested in examining the percentage of caregivers with scores ≥ 15, indicative of major depression to evaluate group differences over 6 months in this subgroup of caregivers with significant symptomatology.

We also evaluated whether caregivers perceived any study benefits using a brief telephone survey at six months. The survey consisted of 9 questions adapted from prior trials [32]: Was study clearly explained? Were you and your relative treated with respect? How much did you benefit from participation? How much did participation help you better understand dementia? How much did participation help you feel more confident dealing with behavioral symptoms? How much did participation make your life easier? How much did participation help enhance your ability to care for your relative? How much did participation in the project help improve your relative’s life? And How much did participation help to keep your relative living at home? Each question was answered by one of three responses: not at all, some, or a great deal. For two additional questions, “did project require too much work/effort,” and “would you recommend this project to others,” caregivers responded either yes or no. We anticipated that both treatment groups would respond favorably regarding their experiences in the study, but that caregivers in the TAP group would indicate greater perceived benefit.

 Finally, to evaluate whether prescribed TAP activities were tolerated by PLWD, caregivers were asked by interventionists at each intervention session if they had used a prescribed activity between sessions; and if yes, whether they observed (not at all, somewhat or very much) three emotional states (interest or pleasure, anxiety or upset, agitation or disruptive behaviors) in the PLWD during the activity.

### Statistical analysis

Based on a pilot study, we assumed 20 % attrition and 80 % power to detect a moderate effect size at 3 months [27]. We set alpha to equal 0.05. Given those numbers, we planned to randomize 250 to TAP or attention controls.

First, chi-squared and t-test tests were used to compare TAP and control dyads on baseline characteristics. Next, an intention-to-treat analysis was performed. For the primary outcome, a sensitivity analysis was conducted using multiple imputation for 44 cases with missing data at the 3-month assessment. Results from the imputation analysis were not substantively different from the complete case analyses, so only the complete case findings are further reported here. For these analyses, all randomized dyads who had 3-month follow-up data were analyzed in the groups to which they were assigned regardless of exposure level to treatment. For main treatment effects, we constructed general linear models to compare each outcome at 3-months between TAP and attention control groups. Baseline value of the outcome measure and baseline MMSE were included as covariates. Also, caregiver sex, race, education, and relationship with PLWD were identified a priori to be included in the analytic models as covariates given their known relationship to the primary and secondary outcomes. Standardized effect sizes (d) were calculated by dividing the differences in covariate-adjusted means by the square root of the mean square error from the model [33].

TAP and control groups were compared on incidence of each type of health event occurring over 6 months using chi-squared tests. The total number of health events experienced by PLWD and caregiver was counted and compared between TAP and control groups with Poisson regression model adjusting for the same covariates as in the main treatment effect models.

To evaluate perceived study benefit, an unadjusted logistic regression was used to evaluate whether TAP caregivers had higher odds of perceiving benefit from the study compared to attention control caregivers. To examine PLWD tolerability of prescribed activities, frequencies and percentages of responses to three emotional states were computed.

Statistical analysis was performed with SAS, version 9.4 (SAS Institute, Cary, NC). All analyses were conducted with the significance level set at *p* < 0.05 and were 2-sided.

## Results

Of 765 inquiries (Figs. 1), 603 (78.8 %) caregivers were screened by telephone of whom 345 (57.2 %) were determined to be eligible. Of these, 69 (20.0 %) were eligible but unwilling to participate and 16 (4.6 %) were found ineligible between the initial screen and the baseline interview appointment. A total of 266 (77.1 %) of the 345 were interviewed at home of whom 250 (72.5 %) were confirmed as eligible and interested in study participation resulting in 124 dyads randomized to receive TAP and 126 randomized to receive the attention control. Forty-four (17.6 %) of the randomized dyads did not complete the three-month follow-up assessment and 74 (29.6 %) did not complete a six-month follow-up. A total of 79 (63.7 %) caregivers in TAP and 61 (48.4 %) caregivers in control were available for the brief six-month follow-up telephone survey to obtain caregiver satisfaction with study participation and perceived benefit.

For dyads lost to follow-up by 3 months, caregivers reported lower cognitive status and poorer quality of life for the PLWD, and a poorer relationship (*p* < 0.05) at baseline compared to retained dyads.

Among randomized dyads, most caregivers were female (*n* = 203, 81.2 %), Caucasian (*n* = 145, 59.2 %) or African American (36.7 %, *n* = 90), with an average age of 65.20 years (SD = 12.64, range = 28–93). Caregivers were mostly adult daughters (*n* = 109, 43.6 %) or wives (*n* = 79, 31.6 %), followed by husbands (*n* = 35, 14.0 %). PLWD were mostly female (*n* = 158, 63.2 %) and Caucasian (*n* = 149, 59.6 %) with an average age of 81.52 years (SD = 8.00, range = 56–99). Descriptive baseline information for the 250 dyads who were randomized are presented in Table 1. (Table 1)

**Table 1:** Characteristics of People Living with Dementia and Their Caregivers by Treatment Arm and Total

Characteristic	Control Group (*n* = 126)	Intervention group (*n* = 124)	Total (*n* = 250)	Χ^2^	t	*p*-value
**People Living with dementia**						
**Age, mean (SD), y**	**80.81 (8.25)**	**82.02 (7.55)**	**81.41 (7.92)**		**1.21**	**0.23**
**Sex, n (%)**				**0.18**		**0.67**
**Male**	**48 (38.10)**	**44 (35.48)**	**92 (36.80)**			
**Female**	**78 (61.90)**	**80 (64.52)**	**158 (63.20)**			
**Race, n (%)**				**1.22**		**0.54**
**White**	**79 (63.71)**	**70 (56.91)**	**149 (60.32)**			
**African American**	**42 (33.87)**	**49 (39.84)**	**91 (36.84)**			
**Other**	**3 (2.42)**	**4 (3.25)**	**7 (2.83)**			
**Education, n (%)**				**0.95**		**0.62**
**≤High school**	**25 (20.00)**	**21 (16.94)**	**115 (46.18)**			
**Some college**	**45 (36.00)**	**41 (33.06)**	**44 (17.67)**			
**≥College**	**55 (44.00)**	**62 (50.00)**	**90 (36.14)**			
**Marital status, n (%)**						
**Married or living as married**	**68 (53.97)**	**64 (51.61)**	**132 (52.80)**	**0.14**		**0.71**
**Other**	**58 (46.03)**	**60 (48.39)**	**118 (47.20)**			
**Living arrangement, n (%)**				**0.05**		**0.82**
**Alone**	**18 (14.29)**	**19 (15.32)**	**37 (14.80)**			
**With caregiver**	**108 (85.71)**	**105 (84.68)**	**213 (85.20)**			
**# of behavioral symptoms, mean (SD)**	**7.87 (2.52)**	**7.76 (2.29)**	**7.82 (2.40)**		**-0.36**	**0.72**
**MMSE score, mean (SD)**	**14.75 (7.49)**	**13.93 (8.16)**	**14.34 (7.82)**		**-0.83**	**0.41**
**Caregivers**						
**Age, mean (SD), y**	**65.44 (12.79)**	**65.29 (12.54)**	**65.37 (12.64)**		**-0.10**	**0.92**
**Sex, n (%)**				**1.43**		**0.23**
**Male**	**20 (15.87)**	**27 (21.77)**	**47 (18.80)**			
**Female**	**106 (84.13)**	**97 (78.23)**	**203 (81.20)**			
**Race, n (%)**				**0.73**		**0.69**
**White**	**76 (61.79)**	**69 (56.56)**	**145 (59.18)**			
**African American**	**42 (34.15)**	**48 (39.34)**	**90 (36.73)**			
**Other**	**5 (4.07)**	**5 (4.10)**	**10 (4.08)**			
**Education, n (%)**				**0.38**		**0.83**
**≤High school**	**58 (46.03)**	**57 (46.34)**	**46 (18.47)**			
**Some college**	**24 (19.05)**	**20 (16.26)**	**86 (34.54)**			
**≥College**	**44 (34.92)**	**46 (37.40)**	**117 (46.99)**			
**Marital status, n (%)**						
**Married or living as married**	**89 (71.20)**	**83 (66.94)**	**172 (69.08)**	**0.53**		**0.47**
**Other**	**36 (28.80)**	**41 (33.06)**	**77 (30.92)**			
**Relationship to patient, n (%)**				**0.42**		**0.52**
**Spouse**	**60 (47.62)**	**54 (43.55)**	**114 (45.60)**			
**Non-spouse**	**66 (52.38)**	**70 (56.45)**	**136 (54.40)**			
**Time of caregiving, mean (SD), y**	**3.87 (2.81)**	**3.94 (3.38)**	**3.90 (3.10)**		**0.17**	**0.86**

Abbreviation: *MMSE* Mini-Mental State Examination

Caregivers reported low depressive symptomatology. At baseline, both groups had a mean score of 5.7 (*p* = 0.99) indicative of mild depressive symptoms, with only 0.05 % in TAP and 0.04 % in the attention control group having scores ≥ 15 (indicative of moderate to severe clinical depression). At three-months, scores remained relatively stable at three months and the percent of caregivers with moderate to severe clinical depression scores did not meaningfully vary between the groups.

### Treatment dose

Of 124 dyads assigned to TAP, 5 (4.0 %), dyads did not participate in any sessions and 119 (96.0 %) had at least one session. Of those, 111 (93.3 %) completed ≥ 4 sessions, considered the minimal treatment threshold with 8 (6.7 %) dyads receiving ≤ 3 sessions. TAP dyads received a mean (SD) of 6.9 (2.1) home sessions with an average of 78.5 (23.0) minutes per session. Of 126 dyads assigned to the control group, 102 (81.0 %) participated in at least one session. Of those, 90 (88.2 %) completed ≥ 4 sessions with 12 (11.8 %) receiving ≤ 3 sessions. Control group dyads received a mean (SD) of 6.5 (2.5) sessions with an average of 54.4 (20.5) minutes per session. Although TAP and control group participants had a similar number of completed sessions (*p* = 0.18), the average length of sessions was shorter by close to 24 min (*p* < 0.0001) for control group versus TAP dyads.

### Tolerability of prescribed activities

**Fig. 2:**
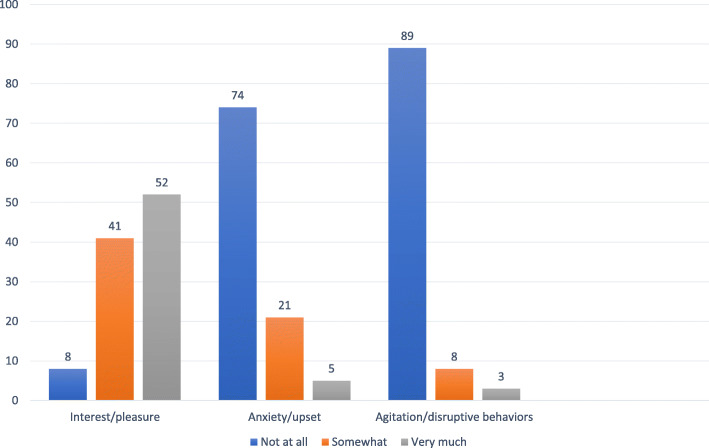
Percent PLWD showing interest, anxiety or agitation with prescribed activities (*n* = 98)

Caregivers reported using the three prescribed activities an average of 9.08 (SD = 8.47) times on their own between sessions in which prescribed activities were introduced, representing an average use of three times each per the three prescribed activities in keeping with OT recommendations. Caregivers also reported that when using prescribed activities, 93.0 % of PLWD showed interest/pleasure somewhat or very much and most had no signs of anxiety (73.6 %) or agitation (88.6 %). Conversely, 7.7 % reported observing that PLWD showed no interest/pleasure, 5.5 % reported presence of anxiety/upset and 3.4 % indicated some form of agitation/disruptive behavior when using the prescribed activities. (Figure 2)

### Outcomes

In unadjusted and adjusted analyses, no statistically significant differences were found from baseline to three months on the primary study outcome variable of frequency by severity of agitation/aggression subscales.

For secondary outcomes, less assistance was needed for TAP PLWD for both IADLs (adjusted mean difference =-0.33; 95 % confidence interval [CI] = (-0.62, -0.05), *p* = 0.02, Cohen’s *d* = -0.33) and ADLs (adjusted mean difference = -0.59; 95 % CI = (-1.15, -0.03), *p* = 0.04, Cohen’s *d* =-0.30) compared to attention controls in unadjusted and adjusted analyses. Also, TAP caregivers reported greater confidence using activities (adjusted mean difference = 3.46; 95 % CI = (0.42, 6.50), *p* = 0.03, Cohen’s *d* = 0.32) and wellbeing (adjusted mean difference = 3.50; 95 % CI = (0.94, 6.06), *p* = 0.01, Cohen’s *d* = 0.39) compared to attention control caregivers (Table 2).

**Table 2:** Comparison of Intervention (*n* = 108) and Control (*n* = 98) Group at 3 Months

Variable	Baseline, mean (SD)		3-month follow-up, mean (SD)		Adjusted Mean Difference Between Groups (95 % CI)	*p*-value	*d*
	**Control Group**	**Intervention Group**	**Control Group**	**Intervention Group**			
**People Living with Dementia Outcomes**							
Agitation/aggression frequency X severity	46.07 (40.27)	45.77 (40.96)	41.04 (40.92)	43.13 (43.32)	3.14 (-4.71, 10.99)	0.43	0.11
IADL dependence	6.96 (1.46)	6.81 (1.56)	7.50 (0.92)	7.16 (1.40)	-0.33 (-0.62, -0.05)	**0.02**	-0.33
ADL dependence	3.67 (2.75)	3.58 (2.67)	4.49 (2.65)	3.88 (2.74)	-0.59 (-1.15, -0.03)	**0.04**	-0.30
**Caregiver Outcomes**							
Confidence using activities	32.80 (11.96)	31.81 (11.85)	34.13 (11.30)	37.18 (12.35)	3.46 (0.42, 6.50)	**0.03**	0.32
Perceived wellbeing	34.27 (6.71)	35.27 (7.05)	38.26 (8.38)	41.73 (9.37)	3.50 (0.94, 6.06)	**0.01**	0.39

Note: Bold signifies significant p values. Abbreviations: *ADL* activities of daily living, *CI* confidence interval, *IADL* instrumental activities of daily living, *SD* standard deviation. d = adjusted difference (intervention – control) in means divided by the square root of the mean square error. All analyses adjusted for baseline value of dependent variable, caregiver sex, race, education, caregiver and patient relationship, and MMSE

### Health-related events

The unadjusted and adjusted Poisson models (not shown) for the total number of health-related events for PLWD and caregivers combined showed that TAP did not statistically significantly reduce these events, although non-significant reductions were observed in TAP that ranged from 18 to 26 % by six months.

More detailed analyses of event types revealed that there were a total of 115 health-related events (PLWD death, hospitalizations, emergency room visits, depression/suicidal ideation; caregiver hospitalizations, depression/suicidal ideation) across both treatment arms. Of the 115 health-related events, 65 (56.5 %) were associated with the PLWD (*n* = 11 deaths; 42 hospitalizations, 10 emergency room visit, and 2 reported depression/suicidal ideation), and 50 (43.5 %) were related to the caregiver (15 hospitalizations, 35 elevated depression scores or suicidal ideation).

As to group differences, there were more PLWD health-related events in the attention control (*n* = 43 events) versus in TAP (*n* = 22 events) for every category (death, hospitalization, depression/suicidal ideation) except emergency room visits in which an equal number of events were reported for both groups. Overall, this represented a 48.8 % improvement in the number of health-related events in TAP for PLWD.

With regard to the number of PLWD with one or more health-related events, there was a statistically significant difference such that 15 PLWD in TAP versus 28 PLWD in attention control) had one or more health-related events (unadjusted *p* = 0.04; adjusted *p* = 0.03). While there were 4 PLWD deaths in TAP versus 7 in attention control, this difference was not statistically significant (unadjusted *p* = 0.37; adjusted *p* = 0.38).

As to caregiver events, there were more hospitalizations in the attention control (*n* = 11 events) versus in TAP (*n* = 4 events), representing a 63.6 % improvement in such events in TAP for caregivers. However, there were slightly more elevated depression/suicidal ideation events in TAP (*n* = 19 events) than in attention control (*n* = 16 events).

As to the number of caregivers, there was a trend towards statistical significance with 3 caregivers in TAP versus 10 in attention control for hospitalizations (unadjusted *p* = 0.06; adjusted* p* = 0.07).

However, for depression/suicidal ideation, slightly more TAP caregivers (*n* = 16) versus control caregivers (*n* = 14) scored ≥ 15 on the PHQ-9 (indicative of moderate clinical depression) or endorsed suicidal ideation (unadjusted* p* = 0.66; adjusted *p* = 0.78). (Table 3).

**Table 3:** Comparison of TAP and Attention Control on Health-related Events: Number of Persons and Events

Category of Event	Total		TAP (*N* = 124)		Attention Control (*N* = 126)		*p*-values = # of persons	
	# Persons	# Events	# Persons	# Events	# Persons	# Events	**Unadjusted*p*-value**	**Adjusted** *p*-value*
**Person living with dementia**								
Death	11	11	4	4	7	7	0.37	0.38
Health related	43	54	15	18	28	36	**0.04**	**0.03**
Hospitalization	34	42	10	13	24	29		
Emergency room	9	10	5	5	4	5		
Depression or suicidal ideation (Proxy report)	2	2	0	0	2	2		
**Caregiver**								
Hospitalizations	13	15	3	4	10	11	0.06	0.07
Elevated depression and/or suicidal ideation	30	35	16	19	14	16	0.66	0.78

Note: Bold signifies significant p value < 0.05; *Adjusted for caregiver race, gender, education, relationship with person living with dementia, baseline Mini-mental Status Examination (MMSE)

### Perceived benefits

TAP and control group caregivers similarly reported better dementia understanding and improved confidence in their caregiving abilities at 6 months (ps > 0.05). However, TAP caregivers compared to attention control group caregivers were more likely to report that participation made life easier (86.1 % vs. 75.0 %, *p* = 0.052), enhanced ability to provide care (97.0 % vs. 85.4 %,* p* = 0.004) and improved PLWD’s life (80.4 % vs. 67.4 %, *p* = 0.048) somewhat or very much.

## Discussion

We conducted a randomized controlled trial to examine the effectiveness of tailoring activities on agitation/aggression as well as on other outcomes that matter to PLWD and caregivers. Contrary to our hypothesis, TAP did not improve agitation/aggression, the main study outcome at 3 months. However, compared to an attention control, in this sample with clinically significant dementia-related agitation/aggression, TAP benefits were evident for PLWD functional dependence, as well as caregiver well-being and confidence in using activity, with less assistance and improved wellbeing and confidence favoring TAP dyads. Benefits for caregivers approximated small to medium standardized treatment effect sizes. Importantly, TAP caregivers tended to report fewer health-related events over six months for PLWD, including deaths and hospitalizations. Also, a trend was found for TAP caregivers to report fewer hospitalizations for themselves compared to controls. These outcomes may result in cost savings which should be a future analytic focus.

Specifically, as it concerns NPS, the main trial outcome, the null treatment effect of TAP was disappointing. This finding is in contrast to other randomized trials of TAP which have shown improvements in a range of behavioral symptoms including agitation [14, 15, 19, 20, 34]. This positive impact of TAP on behavioral symptoms has been reported in trials conducted in the United States [14, 15], Brazil [19, 34], and Australia [20]. The null finding in this trial may be due to several reasons. First, this trial included a sample of PLWD that was determined by a medical team to verify a dementia diagnosis and presence of clinically significant/agitation/aggression. This provided a level of rigor not achieved in other trials resulting in a sample in which all PLWDs had a baseline level of clinically meaningful agitation/aggression-type behaviors. Second, this is the first trial that compared TAP to an attention control group that provided empathy and attention in addition to disease education and home safety tips. The group appeared to be meaningful to caregivers and appeared to have addressed an unmet need for disease education.

Agitation and aggression are among the most complex, problematic and treatment resistant dementia-related symptoms. Under this trial’s test circumstances and for this study sample, there was no impact on these behaviors. Nevertheless, it is possible that some subgroups benefited more than others in this area. Given that other trials have shown behavioral symptom reductions, future analyses are warranted with this same study sample to determine if there were differential treatment effects by gender, relationship or race. Also, future trials with better characterized patient samples should examine TAP effects by disease etiology and disease stage.

 As to functional dependence, TAP instructed caregivers in specific approaches to support activity participation of PLWD including ways to set up the prescribed activities, how to cue and use other communication strategies, and modify the environment to facilitate engagement. Caregivers were also instructed in how to use effective practices to manage basic and instrumental activities of daily living and practiced their use in the presence of the occupational therapist and then independently. Use of strategies may have positively affected functional status by decreasing excess disability or dependence over and above underlying impairments. Although the difference in the number of IADLs and ADLs for which assistance was needed was small between TAP and control groups at 3 months, this difference was significant and also consistent with findings from other TAP trials [15]. The adjusted mean differences of 0.33 and 0.59, respectively, although small, may be clinically significant. It suggests that caregiver assistance was not needed for close to one less daily instrumental/self-care activity. As other studies have shown that increased functional dependence is a predictor of more hours caregiving, hospitalizations and nursing home placements [35], even a small decline in the rate of dependence over time may lessen caregiver burden and improve PLWD quality of life. Trials in Brazil found that TAP resulted in significant quality of life improvements of PLWD as perceived by persons themselves and by their caregivers, and also caregivers perceived quality of life improvements for themselves [19, 34].

The other important finding from this trial was that compared to control group dyads, TAP dyads demonstrated significant health-related improvements: there was a 48.8 % improvement in health-related events for PLWD and a 63.6 % improvement in caregiver hospitalizations in TAP versus controls. The difference in the number of PLWD with hospitalizations in TAP versus control was statistically significant (*p* = 0.03). We did not find however, statistically significant differences in the number of caregivers with major or severe depression [45]. The total number of caregivers by 6 months with elevated scores was a small percentage (0.12 %) of the sample and no large or statistically significant change was observed following treatment in either arm for this subgroup. This suggests that caregivers with clinical depression and/or suicidal ideation need more direct depression-focused interventions to improve on this outcome. This is in contrast to a Brazilian trial which found significant differences such that caregivers in TAP compared to those in usual care had improved quality of life, and decreased depression and burden scores [19].

To our knowledge, there is no other TAP trial that has examined health-related events with one exception. A trial of 160 Veterans found that TAP caregivers compared to those in usual care, reported significantly less pain in PLWD following intervention [15]. That study and the present trial suggest that tailored activities may have far reaching health benefits including reducing incidents of hospitalizations for both PLWD and caregivers that should be further examined in future research studies on TAP and that reduced incidents may result as well in cost savings.

Significantly, caregivers reported that prescribed TAP activities were well tolerated by PLWD with < 5 % demonstrating anxiety and < 3 % agitation “very much” when used at home between treatment sessions. As some, although very few, PLWD were observed as agitated or anxious when engaging in prescribed activities, careful oversight and monitoring appear to be important to address and mitigate PLWD distress if it should occur. TAP is a therapeutic modality and thus, necessitates monitoring.

A critical question remains as to the mechanism by which tailoring activities achieves its positive effects. The data from this trial and others strongly suggests that different mechanisms should be examined to fully understand the pathways by which engaging in tailored activities effects behaviors, function, and other aspects of wellbeing. It may be that tailoring activities to interests and abilities help PLWD remain physically, socially, and psychologically engaged and derive a sense of meaning and purpose. Purpose is an enduring need regardless of disease stage and etiology [36]. There may also be a neurobiological link that should be explored. Remaining engaged in an activity may help people remain physically active, reduce pain, reduce physiological stress and positively affect circadian rhythms [15, 37, 38].

A few study limitations should be noted. First, caregivers who volunteer for a clinical trial may differ in important ways from those who do not volunteer [39]. Enrolled caregivers may be more ready to try new management strategies, such as activities, may have more time to set up and use activities, and may not be as burdened as those who do not volunteer. TAP requires active involvement of caregivers and thus, it is not unexpected that at baseline, those who did not complete the study reported a poorer relationship with PLWD, and lower cognitive status and poorer quality of life of PLWD compared to those who stayed in the study. Second, the measurement of behavioral and psychological symptoms is dependent upon caregiver recall. It is unclear as to the relationship between a caregiver’s mental health and their ratings of behavioral occurrences with some evidence suggesting that depressed caregivers report more behaviors than their counterparts [40]. Caregivers with higher depression and expressed burden may perceive behavioral symptoms as occurring more frequently or severely, but this relationship needs further investigation. Furthermore, some behavioral symptoms may impact caregiver wellbeing more so than others and thus effects their reporting of frequency and severity. There is some evidence to suggest that the number of behaviors being managed versus behavior-type and perceived stress are associated with caregiver burden and depression [41–43]. The evidence is inconclusive and associational, lacking hypothesis-driven analyses or testing of causal mechanisms. While measurement of behaviors symptoms and overreliance on family reports remains a critical issue, we did use the NPI-C which is widely used internationally and shown to be a reliable and valid measure. While caregivers reported behavioral symptoms (versus clinicians), interviewers were trained to explain each behavior using the administration guidelines for the NPI-C. Also, the NPI-C has detailed behaviors for each of its domains, making it the preferred tool in trials such as TAP.

Another limitation may concern disease staging. While we obtained and reported on the cognitive status of PLWD, we were unable to identify etiology and disease stage, a common issue in nonpharmacological trials. Although understanding the role of activity for different disease stages and etiologies may be an avenue for future research, TAP is designed to address and mitigate the functional consequences of any of dementia-type or disease stage. Its tailoring function seeks to assure the “just right fit” between capabilities of PLWD and an activity. Most caregivers overestimate abilities of the person for whom they care [44]. TAP seeks to adjust caregiver communications and the environment and grade activities to match PLWD preserved abilities. That is, TAP seeks to provide a supportive environment such that PLWD is able to function at their best at each disease stage.

Finally, while not a limitation, we did find that contact time differed between the two treatment arms. Future research will need to examine the relationship of contact time on outcomes and dose-response relationships.

Improving quality of life of PLWD is an important treatment goal. Engagement in meaningful activity is an essential and enduring need and caregiver and PLWD report lack of activity engagement as a major concern. Based on the findings from this and other trials, TAP should be considered as a therapeutic approach in dementia care. By enabling engagement in meaningful activities that are structured to match abilities, we show that TAP improves important aspects of quality of life of PLWD. Importantly, TAP improved caregiver wellbeing and reduced caregiver and PLWD health-related events.

This study contributes to the growing body of literature suggesting that individualized activity approaches may address a wide range of clinical symptoms although agitation and aggressive behaviors alluded improvement for this particular study sample. Future research needs to examine program costs, whether caregivers continue to use activities over time and if so, the long-term effects, whether certain dyads benefit more than others, and the underlying mechanisms by which its impacts are achieved.

## Data Availability

The datasets generated and/or analysed during the current study are available from the corresponding author on reasonable request.

## Abbreviations

ADL: Activities of Daily Living
IRR: Incidence rate ratio
MMSE: Mini Mental Status Examination
NPI-C: Neuropsychiatric Inventory – Clinician version
NPS: Neuropsychiatric Symptoms
PHQ-9: Patient Health Questionnaire
PLWD: People living with dementia
TAP: Tailored Activity Program
RCT: Randomized Control Trial
